# SCAN: a cross-species single-cell transcriptome resource for domestic animals—revealing divergent spermatogenesis in avian and mammalian systems

**DOI:** 10.1186/s40104-026-01526-4

**Published:** 2026-11-07

**Authors:** Yuanfang Li, Qianran Wei, Guoxi Li, Ruixing Li, Zhi Li, Xinlei Wang, Liheng Zhang, Yujie Guo, Guirong Sun, Wenting Li, Zhuanjian Li, Yadong Tian, Xiangtao Kang, Zhen Tian, Guohua Wang, Yadong Wang, Qiaoming Liu

**Affiliations:** 1 School of Artificial Intelligence, Henan University, Zhengzhou, 450000 China; 2 School of Medicine and Health, Harbin Institute of Technology, Harbin, 150001 China; 3 Zhengzhou Advanced Research Institute of Harbin Institute of Technology, Zhengzhou, 450000 China; 4 College of Animal Science and Technology, Henan Agricultural University, Zhengzhou, 450046 China; 5 College of Animal Science and Technology, Henan University of Animal Husbandry and Economy, Zhengzhou, 450046 China; 6 School of Computer and Artificial Intelligence, Zhengzhou University, Zhengzhou, 450001 China

**Keywords:** Database, Domestic animals, Single-cell RNA sequencing, Spermatogenesis

## Abstract

**Background:**

Despite the astonishing growth of livestock single-cell transcriptome data, its full potential is severely limited by the lack of specialized functional databases and annotation tools. The ability to systematically integrate, standardize, and deeply interpret livestock single-cell data remains very limited.

**Results:**

This study constructed SCAN, a high-quality resource platform integrating over 2.1 million single-cell datasets from six livestock species (*Sus scrofa*, *Gallus gallus*, *Bos taurus*, *Bos grunniens*, *Ovis aries*, and *Oryctolagus cuniculus*) and a representative model organism (*Danio rerio*). This dataset covers 33 tissue types and 155 cell types, providing differential gene expression profiles for each cell type. Based on this platform, we developed standardized analysis pipelines and visualization tools, and established a livestock marker gene library and a high-quality chicken single-cell annotation reference dataset (CARD). In addition, to demonstrate the utility of the platform, we used SCAN to perform a cross-species single-cell comparison of spermatogenesis between avian and mammalian species. The analysis showed that the early stages of spermatogenesis were relatively conserved across species, whereas differences increased at the later stages; chicken SPT and sperm were significantly enriched in catabolism-related pathways. These observations suggest that the late stages of chicken spermatogenesis may employ a metabolic strategy distinct from that of mammals, but the underlying mechanisms remain to be validated by functional experiments.

**Conclusions:**

This study provides a unified and standardized resource platform (SCAN) for livestock single-cell data, greatly facilitating data access, cell annotation, and downstream biological exploration, thereby enabling downstream biological discovery—as exemplified by the spermatogenesis study presented in this research. The SCAN database is freely and permanently available at http://www.scanimalsdb.bio-zz.cn.

**Supplementary Information:**

The online version contains supplementary material available at https://doi.org/10.1186/s40104-026-01526-4.

## Background

Single-cell RNA sequencing (scRNA-seq) has become a powerful tool for high-throughput transcriptome analysis, providing unprecedented resolution for studying cell states and dynamics [1, 2]. Unlike batch RNA sequencing, scRNA-seq can capture heterogeneity among different cell types within tissues, identify rare but critical cell populations, reveal gene expression patterns in different cell states or developmental stages [3], and analyze intercellular communication within tissues, thereby driving a technological shift in understanding complex biological processes and their underlying mechanisms [4, 5].

With the widespread application of scRNA-seq technology, the quality and quantity of its data have improved significantly in recent years [6]. The vast volume of publicly available scRNA-seq data necessitates effective integration and management, supplemented by strict quality control, data analysis and mining, to ensure the reliability and usability of these datasets. Rapid access to published datasets enables researchers to address scientific questions more efficiently, avoid redundant efforts, and conserve valuable resources [7]. Furthermore, comparing newly generated data with published datasets can help verify research results or propose new biological hypotheses [8]. Public datasets can also facilitate cross-species comparisons [9], offering insights into evolutionary relationships across species. In summary, there is a critical need within the scientific community to leverage bioinformatics tools for the systematic collection, organization and integration of scRNA-seq data, and to establish accessible platforms that support efficient data utilization and in-depth mining.

Currently, there are several databases related to scRNA-seq. For instance, the Human Cell Atlas (HCA) is the largest single-cell database, providing a reference atlas of all cells in the healthy human body [10]. CellMarker 2.0 compiles experimentally validated markers for various cell types in humans and mice [11]. Similarly, PanglaoDB collects single-cell transcriptomic datasets from humans and mice [12]. However, these databases primarily focus on human and mouse species, with a notable lack of datasets related to livestock. Only a few databases contain minimal livestock data, such as the Single Cell Expression Atlas (SCEA), a sub-database of EMBL-EBI [13]. Although SCEA covers a broader range of species, its data volume is relatively limited, and it does not provide data analysis or image visualization function, but focuses on cross-species gene expression. Therefore, to our knowledge, there is currently no scRNA-seq database specifically for livestock that provides standardized bioinformatics analysis and advanced visualization functions, which greatly limits research in this field.

Meanwhile, animal models have become indispensable tools in regenerative medicine [14], not only helping scientists to deepen their understanding of regeneration processes, but also promoting the development of new therapies and technologies [15]. Moreover, driven by growing interest in studying evolutionary relationships among species [16, 17] and the increasing demand for high-quality livestock products [18], research on livestock species has gained widespread attention. In the field of reproductive biology, scRNA-seq provides an ideal approach for elucidating spermatogenesis, a highly conserved but species-specific biological process [19]. Spermatogenesis initiates from spermatogonial stem cells and proceeds through mitotic proliferation, meiosis, and spermiogenesis, ultimately yielding mature sperm [20]. This process relies on intricate interactions between germ cells and somatic cells within the testis, including Sertoli cells (SCs), myoid cells, and Leydig cells [21, 22]. Although the core developmental framework of spermatogenesis is conserved among vertebrates, substantial differences exist between avian and mammalian species in terms of spermatogenic cycle regulation, temperature sensitivity, and key gene regulatory networks [23, 24]. Therefore, cross-species comparisons of testicular tissues at the single-cell level are of critical scientific importance for elucidating the evolutionary adaptations of reproductive strategies and guiding livestock breeding programs. However, the current lack of single-cell databases and marker gene repositories for livestock animals severely hampers the progress of such research.

To address these challenges, we constructed the Single Cell Atlas of Animals (SCAN), a database integrating scRNA-seq data from six livestock species (*Sus scrofa, Gallus gallus*, *Bos taurus*, *Bos grunniens*, *Ovis aries*, and *Oryctolagus cuniculus*) and one model organism species (*Danio rerio*). It encompasses 33 tissues and comprises 2,165,618 cells (as of Dec 20, 2025). Beyond unified data management and access functions, SCAN incorporates standardized analysis pipelines, precise cell annotation procedures, interactive visualization interfaces, cell-type marker gene queries, and a single-cell annotation reference dataset specifically developed for the chicken species, CARD. On this basis, as a demonstration of the platform’s utility, we leveraged the SCAN system to conduct a cross-species comparative study of spermatogenesis in livestock, revealing evolutionary patterns of reproductive development between chickens and mammals, and offering new insights into reproductive adaptation strategies across different species. This platform not only provides critical data support for genetic analysis of reproductive and developmental traits in livestock but also lays a theoretical foundation for molecular breeding practices in domestic animals.

## Methods

### Public data collection and bioinformatics pipeline in SCAN

**Fig. 1:**
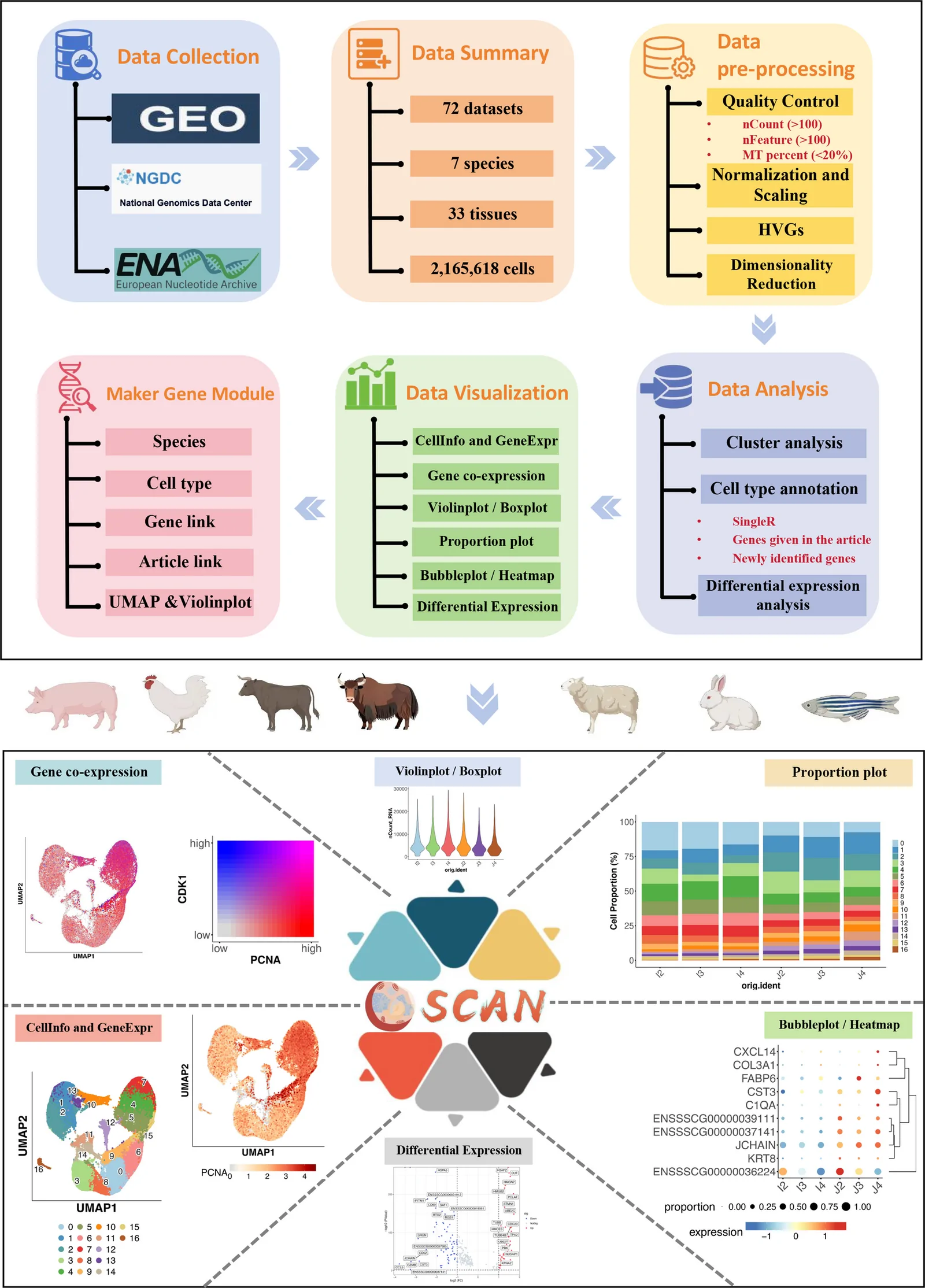
Overview of SCAN’s data collection and processing (above) and six data visualization modules (below)

We searched for scRNA-seq data obtained from three public databases, primarily from the Gene Expression Omnibus (GEO), with additional datasets from the European Nucleotide Archive (ENA) and the National Genomics Data Center (NGDC). We employed a standardized analysis process (Fig. 1) to process the collected datasets, including quality control, batch effect elimination, cell clustering, differential expression analysis, cell type annotation, and cell classification.

### Single-cell data analysis and cluster analysis

The *Seurat* package (v5.3.0) [25] was primarily utilized in the single-cell data analysis. Quality control was first conducted by setting the lower limit for total expression (nCount) and the number of expressed genes (nFeature) per cell to 100, with the upper limit defined as the upper quartile plus three times the interquartile range. Cells with mitochondrial gene proportions exceeding 20% were removed. The 2,000 highly variable genes were identified using the ‘*FindVariableFeatures*’ function, and the ‘*ScaleData*’ function was used to scale their expression. Principal component analysis (PCA) was performed using the ‘*RunPCA*’ function by the *findPC* package (v1.0) [26]. The ‘*RunUMAP*’ and ‘*RunTSNE*’ functions were then applied to further reduce the dimensionality of the harmonized data, resulting in two-dimensional UMAP and t-SNE coordinates. The clustering analysis was performed using the ‘*FindNeighbors*’ and ‘*FindClusters*’ functions from *cluster* package (v0.5.1).

### Cell type annotation analysis

Cell type annotation in SCAN is achieved through a two-step strategy: (i) All data were annotated using the *SingleR* package (v2.2.0) [27]. Depending on the tissue type involved, reference datasets from the *celldex* package (v1.11.1), such as HPCA [28] or Blueprint/ENCODE [29], were selected; (ii) If the article gives the marker genes of the cell type, the cell clusters are annotated as the corresponding cell type again based on the expression of the marker genes of the specific cell type to improve the accuracy of the analysis. The ‘*FindMarkers’* function is then used to identify differential genes between each cell cluster or cell type and the rest of the cells. Finally, the data analysis results are exported as a ShinyApp using the *ShinyCell* package (v2.1.0) [30].

### Database implementation

SCAN is designed with *Django* (https://docs.djangoproject.com/zh-hans/5.1/intro/). For the back end of SCAN, Python is used to handle all data flows, including retrieval. The front-end layout and styling are primarily created using the *Bootstrap* library (https://getbootstrap.com/), while data visualization is developed using the *ECharts* library (https://echarts.apache.org/). Web page interactions are implemented using the JavaScript (https://www.javascript.com/) and the jQuery library (https://jquery.com/). The images displayed on the homepage were created in BioRender (https://BioRender.com/35x2wyg). Nginx (https://www.nginx.com/) is used as a web server to run our application. Data needed for front-end visualizations is stored in XLSX files and processed by a Flask server. All data processing on the server side is handled by Pandas (https://pandas.pydata.org/), a powerful Python package designed for data analysis and processing. It features numerous tools for tasks such as data retrieval and format conversion. For optimal performance and compatibility with the SCAN infrastructure, we recommend using web browsers such as Google Chrome, Firefox, or Microsoft Edge.

### Tissue collection and single-cell library construction

Three healthy roosters were randomly selected from a standard-condition flock and, following euthanasia, their testicular and liver tissues were collected for the subsequent study. The liver and right testis were snap-frozen in liquid nitrogen and stored at −80 °C for RNA extraction, while the left testis was rinsed with PBS and fixed in 4% paraformaldehyde for further RNA localization studies.

Based on our previously published scRNA-seq dataset of chicken testicular tissue [31]. Samples from 20-week-old chickens were selected for cross-species comparative analysis. The data were generated by constructing single-cell transcriptome libraries using the 10X Genomics platform, followed by paired-end sequencing on the Illumina NovaSeq 6000 platform. For comparative analysis, scRNA-seq data of testicular tissue from other species (pig, yak, and sheep) were obtained from the SCAN dataset (GSE174782, GSE225618, and GSE159599), while human data were derived from the GEO dataset (GSE12013) (Table S1).

### Cross-species orthologous gene mapping and transcriptome integration

In order to establish a cross-species comparable expression feature space, the gene symbols or existing Ensembl IDs in the expression matrices of humans, pigs, sheep, yaks, and chickens were first converted into their respective species’ Ensembl gene IDs. The LOC identifier of sheep was also converted with the assistance of Entrez ID. Then, using the hsapiens_homolog_ensembl_gene annotation provided by Ensembl BioMart [32], the genes of non-human species were uniformly mapped to human Ensembl orthologous gene IDs. For each human Ensembl orthologous gene ID, only one non-redundant mapping was retained; duplicate mappings were removed and expression values were not merged. Genes that could not be converted into valid Ensembl IDs, genes that lacked human orthologous annotations, and genes that did not exist simultaneously in the gene sets of all five species were excluded. Then, based on the common mapped gene set and its uniform sorting, the expression matrices of each species were extracted and merged into a cross-species counting matrix by cell. The merged data were then standardized, screened for highly variable genes, scaled, and subjected to principal component analysis. Then, species origin was used as a correction variable for Harmony integration [33]. Finally, the integrated data were used to construct nearest neighbor graphs, cell clustering, and UMAP visualization.

### Differential expression analysis and enrichment analysis

Differentially expressed genes were identified using the ‘*FindAllMarkers*’ function in the *Seurat* package (v5.3.0) with the Wilcoxon rank-sum test. The filtering thresholds were set as *P*_adj_ < 0.05, log_2_|FC| > 0.25, and *pct.1* > 0.1. GO and KEGG pathway enrichment analysis of the differentially expressed genes was performed using the R package *clusterProfiler* (v4.8.3).

### Correlation analysis

Correlation analysis was based on the normalized mean gene expression across all cells within each species, and Pearson correlation coefficients were calculated between every pair of species or cells.

### Cell cycle analysis and cell–cell communication analysis

Cell cycle analysis was performed using Seurat’s built-in cell cycle feature gene sets, which include markers for the S phase and G2/M phase. Cell–cell communication analysis was conducted using the *CellChat* R package (v2.1.1) [34].

### Pseudotime trajectory analysis

Pseudotime trajectory analysis was performed using the *Monocle3* (v1.3.4) R package. To compare the differentiation dynamics between humans and pigs during the spermatocyte stage, two branching trajectories were established within the SPC cell population, and gene expression changes along pseudotime were modeled using the ‘*fit_models*’ function. Genes exhibiting significantly opposite expression trends between the two branches were identified by comparing the coefficients of the regression models and ranking them based on the magnitude of the difference in regression coefficients.

### RNA extraction and real‑time quantitative PCR validation

Total RNA was extracted from testicular tissues using the TRIzol reagent (Invitrogen Life Technologies, Shanghai, China), reverse‑transcribed, and subjected to SYBR Green‑based real‑time quantitative PCR to detect the expression levels of target genes. The *GAPDH* gene served as the internal control, and relative gene expression was calculated using the 2^−ΔΔCt^ method [35]. The primer sequences are provided in Table S2. Three technical replicates were performed for each sample. Differences between the two groups were analyzed using the *t*-test in the GraphPad Prism software (version 9.0.0), with significance levels set at ^*^P < 0.05, ^**^P < 0.01, and ^***^P < 0.001.

### Fluorescence in situ hybridization

Fixed testicular tissues were embedded in paraffin, sectioned, and permeabilized, followed by overnight hybridization with fluorescence‑labeled specific probes. After washing off non‑specifically bound probes, nuclei were counterstained with DAPI. Sections were mounted and imaged under a Nikon fluorescence microscope (Nikon Corporation, Tokyo, Japan) to analyze the spatial expression patterns of target RNAs in the tissue.

### Cross-species bulk RNA-seq analysis

Bulk RNA-seq datasets from muscle satellite cells in *Homo sapiens*, *Mus musculus*, *Sus scrofa*, *Bos taurus*, and *Gallus gallus* were downloaded from the GEO database for cross-species analysis (Table S1). The data analysis workflow included: normalization of raw data using the TPM method; cross-species gene ortholog conversion using the *biomaRt* package (v2.59.1); construction of a hierarchical clustering tree based on Euclidean distances to quantify transcriptomic similarities among species; and dimensionality reduction via PCA with visualization of species-level transcriptomic heterogeneity using the first two principal components.

### CARD dataset construction

Bulk RNA-seq datasets from various primary chicken cell types were collected from the GEO database. Expression values of all samples were uniformly converted to TPM format, integrated into a standardized gene expression matrix, and packaged as a *SummarizedExperiment* object. This data structure is fully compatible with the input requirements of the *SingleR* package (v2.4.0), enabling direct application in automated annotation pipelines for scRNA-seq data. Marker genes used in this study were primarily selected based on previously reported literature [36, 37]. Finally, the Accuracy and Kappa metrics between the annotation results of each reference dataset and the manual annotations were calculated using the *confusionMatrix* function from the *caret* package (v7.0-1) to evaluate the precision of the CARD dataset.

## Results

### Design and general description of the database

As a user-friendly web server, SCAN provides comprehensive functionality, including single-cell sequencing data deployment, dataset search and data analysis, marker gene search, and a detailed user manual. The SCAN database contains 72 datasets, primarily from six domestic animals and one model organism (zebrafish). The “Data” page summarizes key information such as species, tissue, sequencing technology, publication details, cell type, and marker genes. The Data Analysis page covers 2,165,618 cells from these 72 datasets, involving 155 cell types and encompassing 7 species and 33 different tissues (Fig. 1). Based on the processed data, we provide standardized visualization results in the “Data Analysis Link” module. Most of the results have been precisely annotated for cell types according to the original literature (if available), and users can directly download them for subsequent verification. Furthermore, the “Marker Gene” module not only lists the relevant marker genes reported in the literature, but also details their specific expression patterns in different cell types within the tissue.

### Statistics of SCAN

**Fig. 2:**
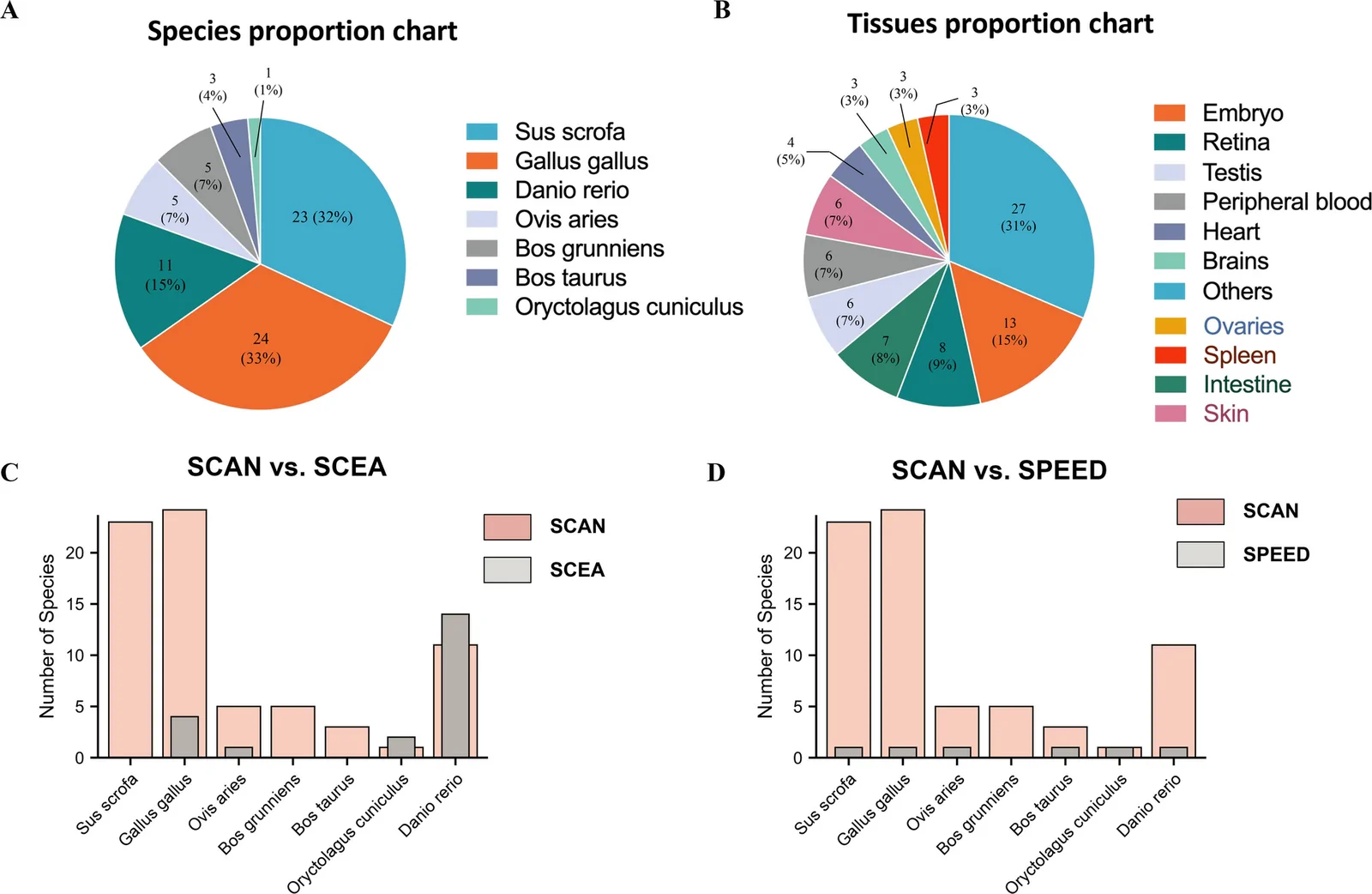
Statistics of the SCAN database and comparison with peer resources. **A** Composition distribution of species included in the SCAN database. **B** Distribution of tissue/organ types covered by the SCAN database. **C** Comparison of livestock species coverage between the SCAN and SCEA databases. **D** Comparison of livestock species coverage between the SCAN and SPEED databases

The SCAN database includes a total of seven species, comprising six domestic animal species: *Sus scrofa*, *Gallus gallus*, *Bos taurus*, *Bos grunniens*, *Ovis aries*, and *Oryctolagus cuniculus* as well as one model animal species, *Danio rerio*. Among these, the species with the largest number of datasets were *Sus scrofa* and *Gallus gallus*, with 23 and 24 datasets respectively, together accounting for 65% of the total. These were followed by *Danio rerio* (11 datasets), *Ovis aries* (5), *Bos grunniens* (5), *Bos taurus* (3), and *Oryctolagus cuniculus* (1) (Fig. 2A). We also analyzed the tissue/organ distribution across the seven species. The majority of studies focused on embryo (13 datasets, 15%), retina (8, 9%), intestine (7, 8%), peripheral blood (6, 7%), testis (6, 7%), and skin (6, 7%), followed by heart (4, 5%), brain (3, 3%), ovaries (3, 3%), and spleen (3, 3%) (Fig. 2B). Next, we compared the 7 animal species in the SCAN database with two other databases that cover a wider range of species. The SCEA database comprises only four *Gallus gallus* datasets, one *Ovis aries* dataset, and two *Oryctolagus cuniculus* datasets (Fig. 2C). In contrast, SCAN includes *Sus scrofa*, *Bos grunniens*, and *Bos taurus* datasets, whereas SCEA does not. The SPEED database contains data from five species: *Sus scrofa*, *Gallus gallus*, *Ovis aries*, *Bos taurus*, and *Oryctolagus cuniculus* (Fig. 2D). SCAN adds a dataset on *Bos grunniens* compared to SPEED, and the number of datasets for domestic animal species in SCAN is significantly higher than in both the SCEA and SPEED databases.

### User-friendly data search and single-cell visualization module

To make it easier for users to find datasets of interest, the SCAN database offers two search methods: (i) The homepage features quick search buttons categorized by species, allowing users to filter datasets based on their species of interest (Fig. S1A); (ii) All datasets in the SCAN database are organized on the “Data” page, where users can filter datasets by six criteria: publication date, species, tissue, platform, sequencing technology, and reference database (Fig. S1B). Through these filtering methods, users can obtain relevant datasets along with corresponding article information (e.g., article title, abstract, publication date). Additionally, users can click the “Data analysis link” to view a visual representation of the datasets (Fig. S1C).

The single-cell data analysis visualization page, built on *Shiny*, offers six modules for visualizing analysis results, along with downloads of “Seurat objects” and statistical graphs (Fig. S2A). It is worth emphasizing that, to ensure annotation accuracy, the cell types for most of the data have been precisely annotated based on the marker genes or data labels provided in the original literature. For data where the original literature did not provide marker genes, we performed annotations using the Human Primary Cell Atlas Data (HPCA) or Blueprint/ENCODE datasets provided by *SingleR*. The user can intuitively view the distribution of cell type information and gene expression (Fig. S2B), explore gene co-expression patterns (Fig. S2C), compare expression differences across samples or cell clusters using violin plots/box plots (Fig. S2D), examine the proportional distribution of each cell type within samples (Fig. S2E), analyze gene expression across cell clusters with bubble plots/heatmaps (Fig. S2F), and filter key genes via volcano plots or data tables in the differential expression module (Fig. S2G). All charts support highly customizable and interactive operations, allowing users to download analysis results and statistical graphs with one click, meeting the needs for in-depth exploration.

### Marker gene module

In order to fill the gap in the summary of livestock marker genes, we compiled a list of marker genes corresponding to different cell types, based on a thorough manual review of the literature. The cell type marker list consists of the following columns: (i) Species, (ii) Cell type, (iii) Marker genes, (iv) Article title, (v) Data name, (vi) UMAP, and (vii) Violinplot (Fig. S3A). Additionally, when the user clicks on a gene name, they are redirected to the gene’s page on the NCBI website (Fig. S3B). Similarly, clicking on an article name takes the user to the article’s PubMed page (Fig. S3C). This feature enables users to access detailed information more easily. In addition, we also plotted the UMAP and violin plots of this maker gene in the corresponding data, allowing users to examine its expression across cell types (Fig. S3D).

### Application of SCAN—cross-species spermatogenesis analysis

**Fig. 3:**
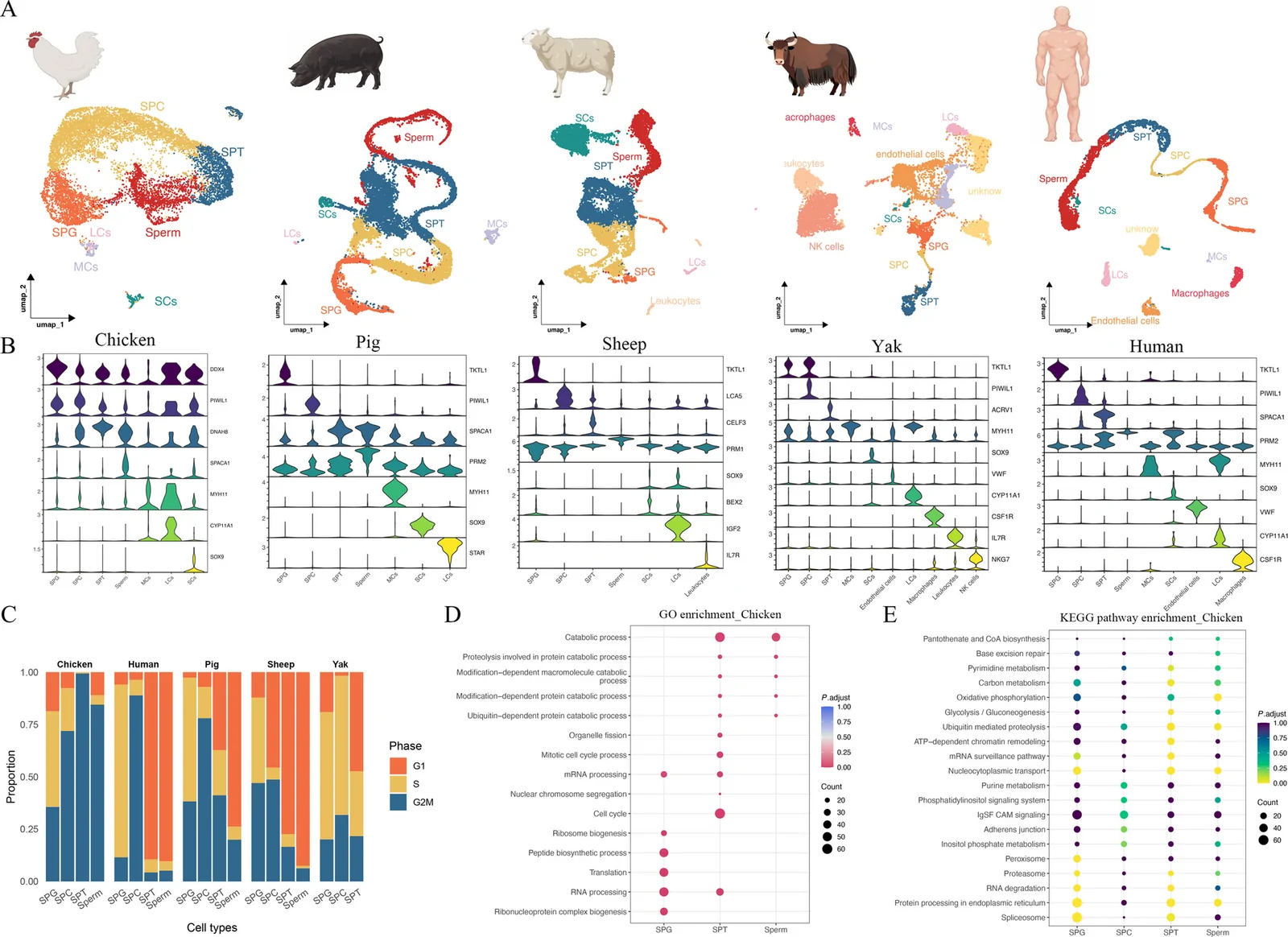
Single-cell transcriptomic profiles of testis from five species based on SCAN-integrated datasets. **A** UMAP projection of scRNA-seq datasets from the testis of chicken, human, pig, sheep, and yak. Germ cells and somatic cells are color-coded consistently with their respective cell type labels. For example, SPG (orange-yellow), SPC (yellow), SPT (blue), and sperm (red). **B** Violin plots showing expression of selected germ cell and somatic cell markers. For example, SPG (*TKTL1*, *DDX4*), SPC (*PIWIL1*, *LCA5*), SPT (*SPACA1*, *CELF3*, *ACRV1*, *DNAH8*), sperm (*PRM1*, *PRM2*), SCs (*SOX9*), MCs (*MYH11*), LCs (*IGF2*, *CYP11A1*, *STAR*), leukocytes (*IL7R*), NK cells (*NKG7*), endothelial cells (*VWF*), and macrophages (*CSF1R*). **C** Cell cycle phase proportions of four germ cell types across the five species. **D** GO enrichment analysis of germ cells in chicken. **E** KEGG enrichment analysis of germ cells in chicken. Sperm cells were not identified in the yak dataset

To further leverage SCAN as a valuable resource for biological investigation, we performed a cross-species analysis of testicular tissues. This analysis integrated datasets from pigs (*Sus scrofa*), yaks (*Bos grunniens*), and sheep (*Ovis aries*) available in SCAN with our own sequencing data from chickens (*Gallus gallus*) and publicly available human (*Homo sapiens*) data. Following quality control and filtering, we obtained transcriptomes for a total of 53,504 high-quality cells across the five species. The final dataset had a mean of 10,701 cells per species, a median of 2,140 unique molecular identifiers (UMIs) per cell, and low percentages of mitochondrial UMIs (Table S3). We identified the major germ cell types involved in the continuous proliferation and differentiation pathways of spermatogenesis across all species: spermatogonia (SPG), spermatocytes (SPC), spermatids (SPT), and sperm. We also characterized the somatic cells of the testicular tissues, notably Sertoli cells (SCs), which support spermatogenesis, as well as other cell types, including Leydig cells (LCs), myoid cells (MCs), endothelial cells, NK cells, leukocytes, and macrophages (Fig. 3A). We leveraged the marker gene library from the SCAN database to perform rapid and accurate cell annotation. For example, we selected genes *TKTL1* and *PIWIL1* from the SCAN database as marker genes for SPG and SPC cells, respectively (Fig. 3B). Cellular proportion analysis revealed (Fig. S4, Table S3) that the four germ cell types (SPG, SPC, SPT, and sperm) were relatively evenly distributed in chickens and pigs, with germ cells accounting for over 96% of the total cell population. In humans and sheep, germ cells comprised approximately 73%−78%, whereas in yaks, germ cells represented only about 13%, and somatic cells accounted for as high as 87%. These differences may not only reflect inherent biological characteristics of the species, but may also be influenced by technical discrepancies across the original studies, such as tissue sampling strategies and dissociation protocols.

In addition, we further focused on the four germ cell types and conducted cell cycle analysis (Fig. 3C). The results showed that SPC across different species was predominantly in the G2/M phase, indicating that the initiation and progression of meiosis (which occurs during the G2/M phase) represent a highly conserved core event in spermatogenesis. In contrast, SPG were mainly in the S phase, consistent with the universal role of SPG proliferation across species. Notably, human SPG showed the highest proportion in the S phase. While SPT and sperm in humans, pigs, sheep, and yaks were primarily in the G1 phase, these two cell types in chickens were mainly in the G2/M phase, highlighting a distinct cell-cycle regulation in avian spermatogenesis.

GO functional enrichment analysis (Fig. 3D and Fig. S5A) showed that the enrichment patterns of SPG and SPC were similar across species, as were those of SPT and sperm. Specifically, SPG and SPC in all five species were mainly enriched in GO terms, such as ribonucleoprotein complex biogenesis and RNA processing, with yaks additionally enriched in metabolic processes, like the DNA metabolic process. Moreover, SPT and sperm in the four mammalian species were primarily enriched in GO terms such as germ cell development, spermatid differentiation, and cilium movement, while in chickens, SPT and sperm were mainly enriched in metabolism-related GO terms such as catabolic process. Furthermore, KEGG enrichment analysis revealed (Fig. 3E and Fig. S5B) that SPG and SPC were mainly enriched in pathways such as the Ribosome pathway and the Cell cycle pathway across five species, with yaks showing more pronounced enrichment in the DNA replication pathway. SPT and sperm were primarily enriched in metabolic pathways such as Carbon metabolism and Purine metabolism among the five species. While enriched in chickens, humans, and pigs, the ubiquitin-mediated proteolysis pathway showed species-specific activity patterns: in SPT/sperm (chickens), SPG/SPC (humans), and across all germ cell stages (pigs).

### Chicken testicular cells showed the lowest correlation with mammalian species

**Fig. 4:**
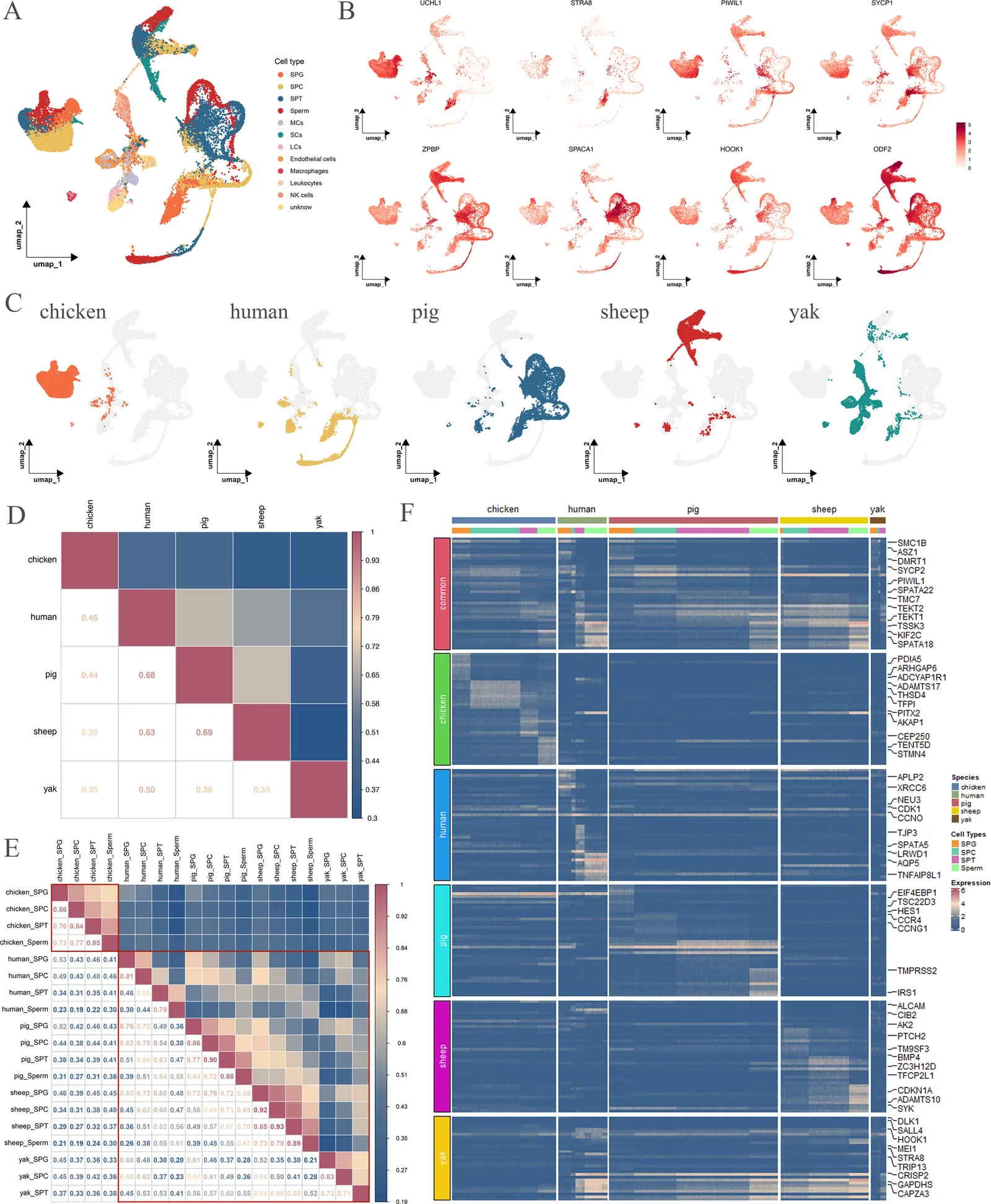
Cross-species scRNA-seq analysis of testicular tissues using SCAN-integrated data. **A** Integrated UMAP visualization of testicular cells from five species. **B** Expression of selected germ cell marker genes projected onto the UMAP, exemplified by SPG (*UCHL1*, *STRA8*), SPC (*PIWIL1*, *SYCP1*), SPT (*SPACA1*, *ZPBP*), and sperm (*HOOK1*, *ODF2*). **C** UMAP plots separated by species. **D** Correlation analysis of testicular tissues across the five species. **E** Correlation analysis of distinct germ cell types (SPG, SPC, SPT, and sperm) in testicular tissues across the five species. **F** Heatmap of conserved and species-specific genes across distinct germ cell types (SPG, SPC, SPT, and sperm) in the five species

To compare the similarities and differences across species, we integrated all cells from different species (Fig. 4A). In Fig. 4B, we displayed the expression patterns of marker genes such as *UCHL1* and *SYCP1*, which validated the accuracy of our classification. Furthermore, we analyzed each species separately and found that the four mammalian species exhibited substantial overlap in SPG, whereas they began to separate in SPC (Fig. 4C). Notably, no overlap was observed between chicken germ cells and those of other mammals. To further investigate the relationships among different cell types across species, we performed correlation analysis (Fig. 4D). Overall, chickens show relatively low correlation with the comparative mammalian species (*r* < 0.46). Interestingly, in testicular tissue, chickens still exhibit a higher correlation with humans than with pigs, sheep, and yaks. At the cellular level, the four types of chicken germ cells (SPG, SPC, SPT, and sperm) also display relatively high expression similarity with their human counterparts (*r* < 0.53) (Fig. 4E). Nevertheless, these similarities do not alter the fact that chickens remain evolutionarily distant from mammals overall (*r* < 0.53). Moreover, germ cell correlations across species followed a consistent pattern: similarity was highest in SPG (*r* > 0.46) and gradually decreased as spermatocytes differentiated, with sperm exhibiting the lowest correlation (*r* > 0.3). This pattern was corroborated by UMAP visualization, where SPG populations from the four mammals largely overlapped, while SPC began to separate (Fig. 4C).

To identify conserved and species-specific genes during spermatogenesis across different species, we integrated differentially expressed genes among germ cell types (SPG, SPC, SPT, and Sperm) from each species using the screening criteria of *P*_adj_ < 0.05, *log*_*2*_*|FC|* > 0.25, and *pct.1* > 0.1. The results showed that 7,012, 10,471, 6,927, 6,336, and 3,629 differentially expressed genes were identified in chickens, humans, pigs, sheep, and yaks, respectively (Fig. S6A). The 1,545 genes conserved across all five species (Fig. S6B) were predominantly enriched for the GO terms nuclear chromosome segregation and ribonucleoprotein complex biogenesis, and for the pathways of Nucleocytoplasmic transport, Protein processing in endoplasmic reticulum, and Cell cycle (*P*_adj_ < 0.05). Furthermore, enrichment analysis of species-specific genes showed that chickens were primarily enriched in the extracellular matrix organization and axon development GO term, as well as the cAMP signaling pathway and the Calcium signaling pathway (*P*_adj_ < 0.05), sheep were mainly enriched in the ameboidal-type cell migration GO term and the Neuroactive ligand-receptor interaction pathway (*P*_adj_ < 0.05), humans were predominantly enriched in the axon development GO term and the Calcium signaling pathway (*P*_adj_ < 0.05) (Fig. S6C and D), pigs were mainly enriched in the Insulin signaling pathway (*P*_adj_ < 0.05), and yaks were mainly enriched in the actin myofibril assembly GO term (*P*_adj_ < 0.05). We found that humans and chickens share numerous overlapping GO terms. To further reveal conserved and species-specific expression patterns across cell types, we performed separate Venn diagram analyses of differentially expressed genes for each cell type (Fig. S6E) and examined the expression patterns presented in the heatmap (Fig. 4F). The results indicated that in SPG, *SMC1B* and *DMRT1* genes were shared among all five species; in SPC, *SYCP2* and *SPATA22* genes were shared among all five species; in SPT, *TEKT1* and *TEKT2* genes were shared among all five species; and in sperm, *KIF2C* and *SPATA18* genes were shared among all five species (with sperm not detected in yaks) (Table S4). We also identified a large number of genes with divergent expression patterns between chickens and mammals. For example, genes such as *KPNA4* and *TRAF3IP1* are highly expressed in chicken sperm, whereas genes including *TSSK3* and *GLUL* show high expression levels in mammalian sperm (Fig. S6F and G). In addition, functional enrichment analysis of the conserved genes showed stage-specific associations: SPG genes were primarily involved in ribosome biogenesis, nucleocytoplasmic transport, RNA processing, and ncRNA processing; SPC genes were enriched in nuclear chromosome segregation; SPT genes in cilium assembly; and sperm genes in translational initiation (Fig. S6H and I).

**Fig. 5:**
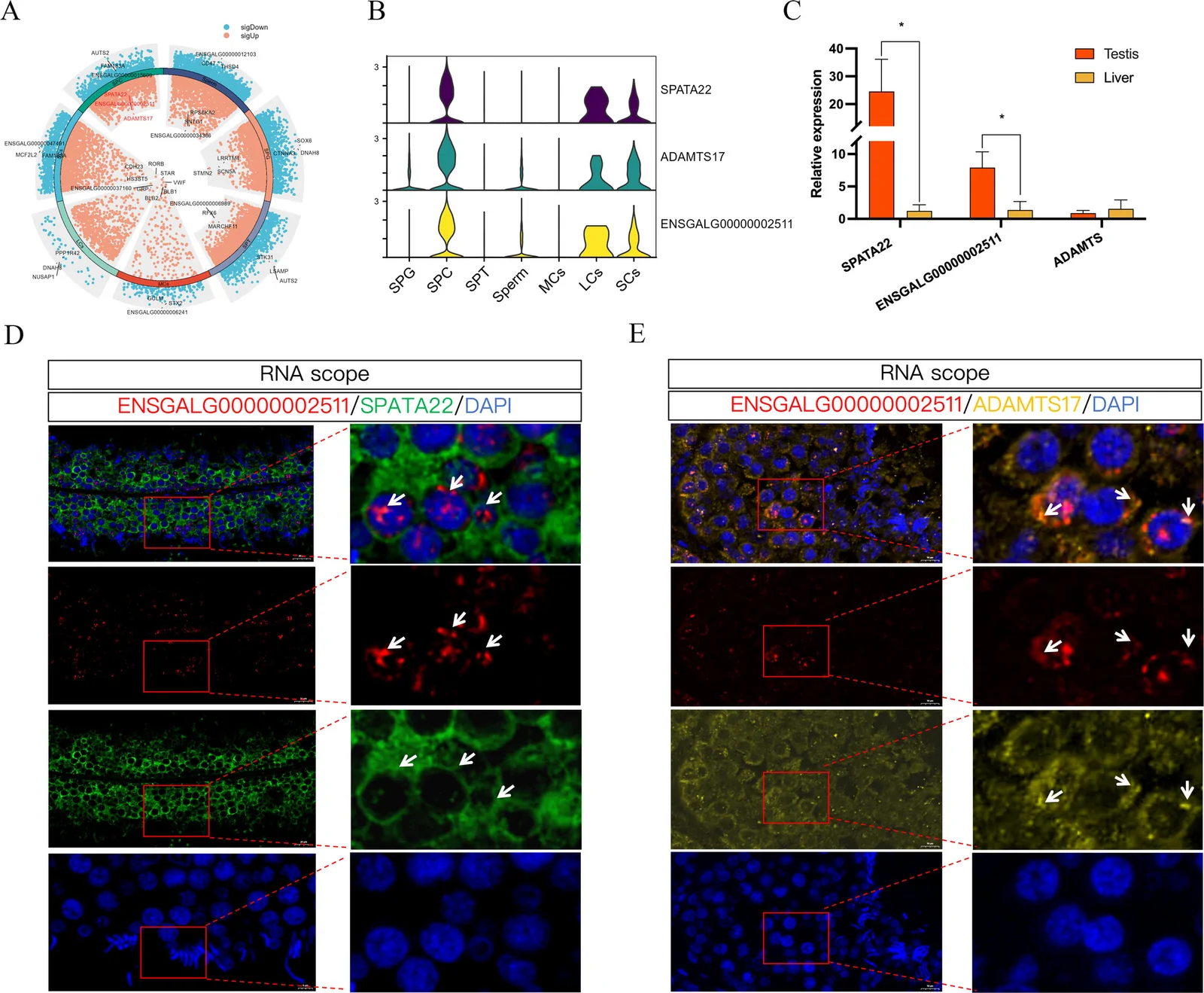
Identification of SPC marker genes in chicken testis. **A** Volcano plot of differentially expressed genes across distinct cell types in chicken testis. **B** Violin plot of highly expressed genes in SPC of chicken testis. **C** mRNA expression levels of *SPATA22*, *ADAMTS17*, and *ENSGALG00000002511* in testicular tissues, liver tissue served as the control. Statistical significance is denoted as follows: ^*^P < 0.05. **D** Double fluorescent in situ hybridization of testicular SPC marker *ENSGALG00000002511* (red) and *SPATA22* (green), nuclei are stained with DAPI (blue). Scale bar: 5 μm. **E** Double fluorescent in situ hybridization of testicular SPC marker *ENSGALG00000002511* (red) and *ADAMTS17* (yellow), nuclei are stained with DAPI (blue). Scale bar: 5 μm

Given the species specificity of chickens, we further conducted a separate analysis of differentially expressed genes in each cell type of chickens (Fig. 5A). The results revealed significant differential expression of *ADAMTS17*, *SPATA22*, and *ENSGALG00000002511* in SPC (Fig. 5B). Among these, *ENSGALG00000002511* corresponds to the human homologous gene *SYCP1*. Given that *SYCP1* serves as a marker gene for human SPC, we employed it as a positive control in the fluorescence in situ hybridization (FISH) experiment. These experimental results demonstrate that *SPATA22* is highly expressed in both chicken testicular tissue and SPC cells (Fig. 5C and D), and *ENSGALG00000002511* is primarily expressed in the nuclei of SPC. In contrast, *ADAMTS17* is specifically enriched in SPC cells despite its low expression in bulk testicular tissue (Fig. 5C and E). The discrepancy between *ADAMTS17* expression at the tissue level and the cellular level further underscores the need for single-cell resolution analysis.

### Chicken testicular tissue possesses a unique cell–cell communication network

**Fig. 6:**
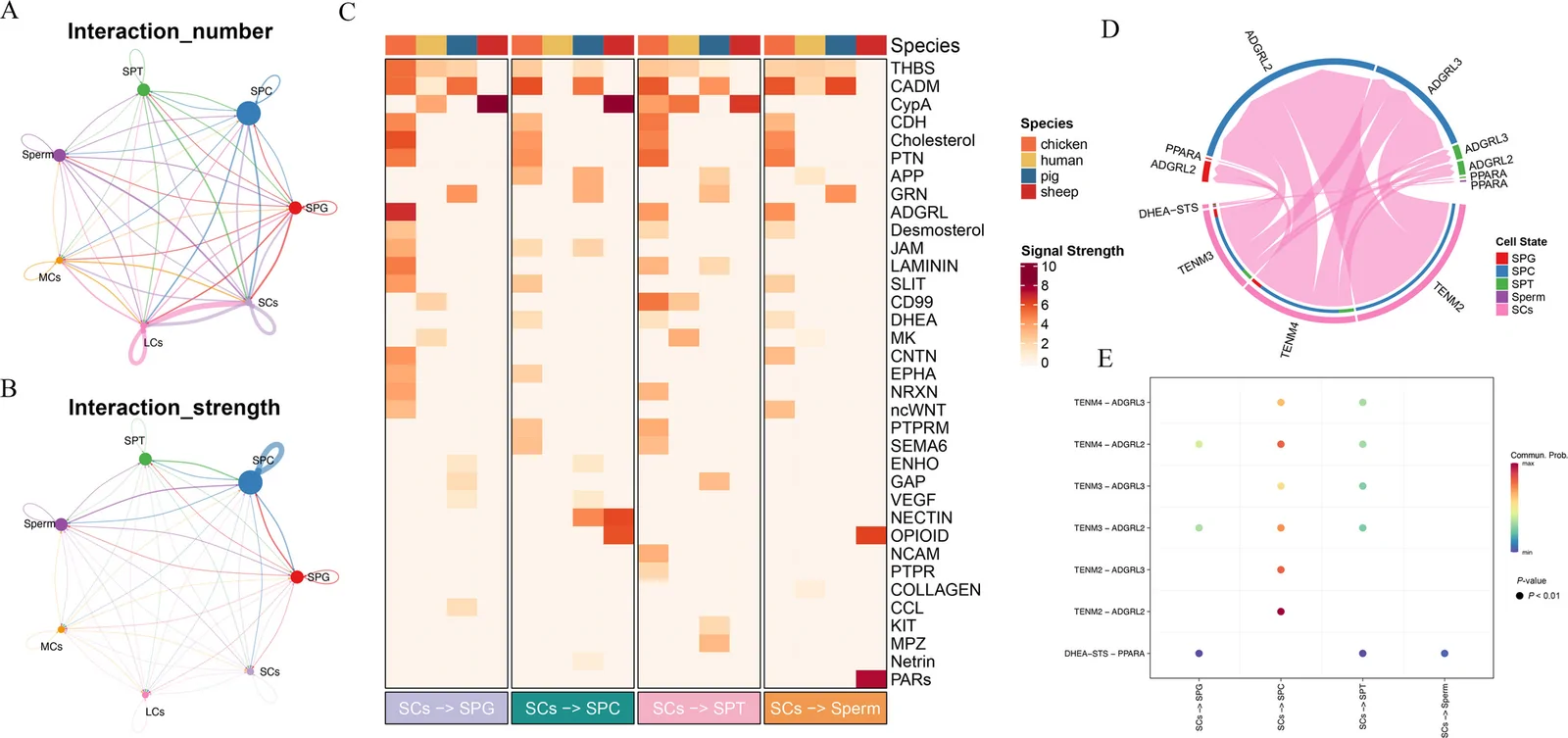
Analysis of cell–cell communication. **A** Analysis of the number of cell–cell communication networks among different cell types in chicken testicular tissue. **B** Analysis of the strength of cell–cell communication networks among different cell types in chicken testicular tissue. **C** Comparative analysis of communication pathways between Sertoli cells and germ cells in testicular tissues of chicken, human, pig, and sheep. **D** and **E** Analysis of ligand-receptor pairs involved in ADGRL and DHEA signaling pathways between Sertoli cells and germ cells in chicken testicular tissue

To further identify differences in somatic-germ cell communication across species, we performed cell–cell communication analysis (Fig. 6A–B and Fig. S7A–B). Given the critical supportive role of Sertoli cells in testicular tissue, we focused on cell communication between Sertoli cells and four types of germ cells (no relevant signals were detected in yaks). Here, Sertoli cells were designated as signal senders (expressing ligands), while germ cells were designated as signal receivers (expressing receptors). The analysis results showed that in chickens, Sertoli cells exhibited strong and extensive communication with the other four types of germ cells (Table S5). Among these, CDH signaling and Cholesterol signaling were observed explicitly in chickens (Fig. 6C). In sheep, Sertoli cells also exhibited strong communication with germ cells, although the number of signaling pathways was relatively limited, primarily focusing on CypA signaling. In contrast, communication between human Sertoli cells and germ cells was weaker (Fig. 6C). Furthermore, THBS signaling and CADM signaling were mainly detected in communication between Sertoli cells and germ cells in chickens, humans, and pigs. Notably, ADGRL and DHEA signaling were uniquely observed in chickens (Fig. 6C and Fig. S7C–D), and interestingly, both of which are associated with the cAMP signaling pathway. We further analyzed the receptor-ligand pairs involved and found that pairs such as TENM2-ADGRL2 were present in signals sent from Sertoli cells to SPCs, while DHEA-STS-PPARA was present in signals sent from Sertoli cells to SPG, SPT, and sperm (Fig. 6D–E and Fig. S7E).

### Divergence between human and pig spermatogenesis occurs at the spermatocyte stage

**Fig. 7:**
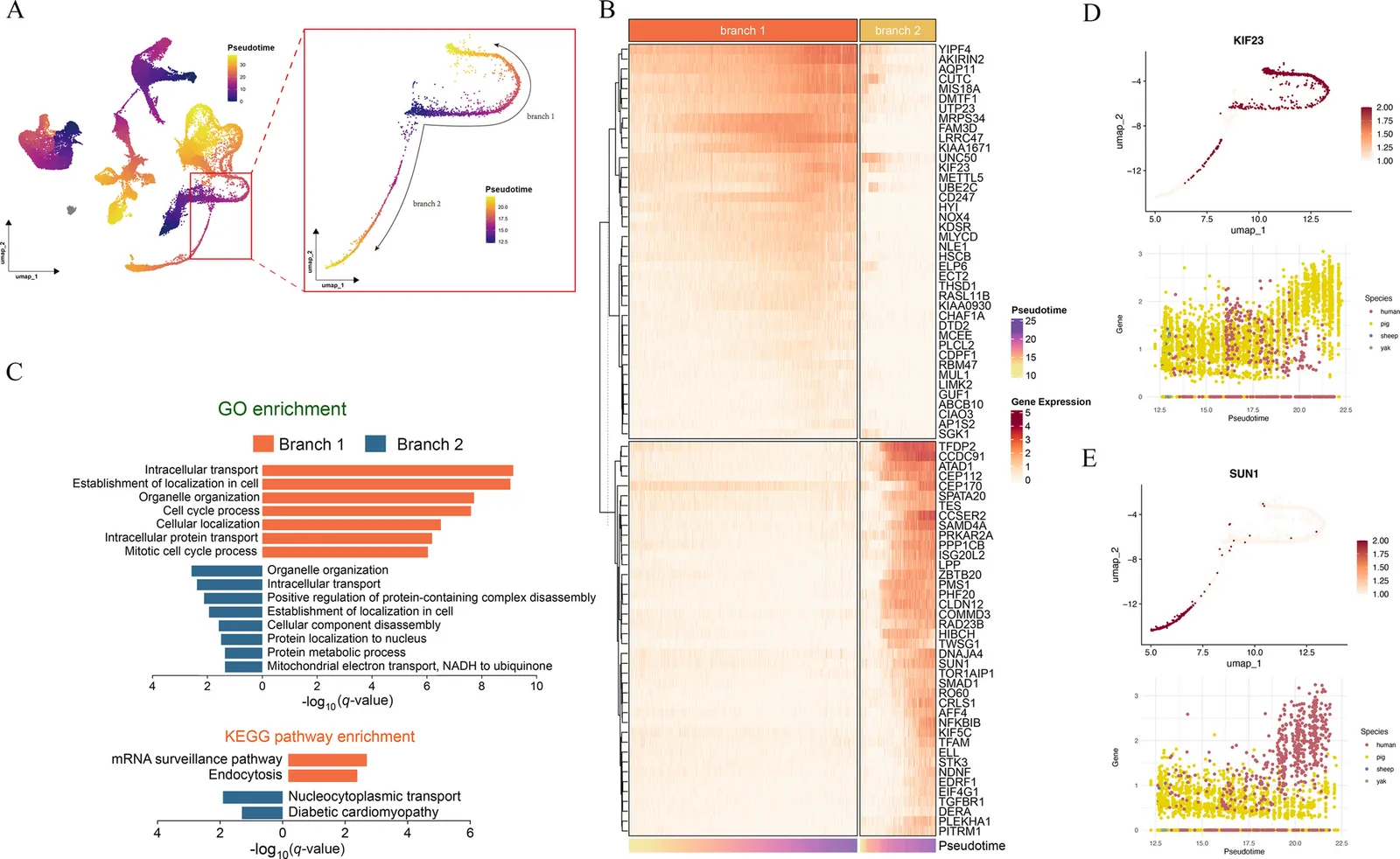
Trajectory analysis of differentiation. **A** Pseudotime analysis highlighting the differentiation trajectory from SPG to SPC in human and pig, primarily divided into two branches: Branch 1 and Branch 2. **B** Heatmap of gene expression dynamically regulated along the Branch 1 and Branch 2 trajectories. **C** GO and KEGG pathway enrichment analysis of feature genes associated with Branch 1 (orange-yellow) and Branch 2 (blue). **D** Dynamic expression changes of the key Branch 1 gene *KIF23* along pseudotime. **E** Dynamic expression changes of the key Branch 2 gene *SUN1* along pseudotime

To elucidate the divergence point across these species during spermatogenesis, we performed trajectory analysis on cross-species integrated data (Fig. 7A and Fig. S8). The results revealed that with SPG as the starting point, the overlapping trajectory primarily consisted of human and pig cells, while cells from other species were more dispersed. Therefore, we focused our analysis on deciphering the differentiation drivers in humans and pigs. The findings indicated that SPG from humans and pigs exhibited highly overlapping distributions, suggesting conservation in early developmental stages. However, significant divergence began at the SPC stage, ultimately resulting in the formation of two main branches: Branch 1, predominantly composed of pig cells, and Branch 2, mainly consisting of human cells (Fig. 7A). To investigate the driving mechanisms behind this divergence, we further identified genes that exhibited changes in expression along the two branches. Two gene clusters were found to jointly promote this differentiation process: 507 genes were upregulated along Branch 1 and downregulated along Branch 2 (Fig. 7B, Table S6), primarily enriched in intracellular transport-related GO terms and the mRNA surveillance pathway (Fig. 7C), including genes such as *KIF23* (Fig. 7D). Conversely, 251 genes were upregulated along Branch 2 and downregulated along Branch 1 (Fig. 7B, Table S6), primarily enriched in GO terms related to organelle organization and the nucleocytoplasmic transport pathway (Fig. 7C), including *SUN1* (Fig. 7E). These key gene clusters, which drive the divergence between humans and pigs and have been identified at the spermatocyte stage, provide molecular-level insights into interspecies differences during spermatogenesis.

### Cross-species bulk RNA-seq analysis reveals significant differences between chickens and mammals

**Fig. 8:**
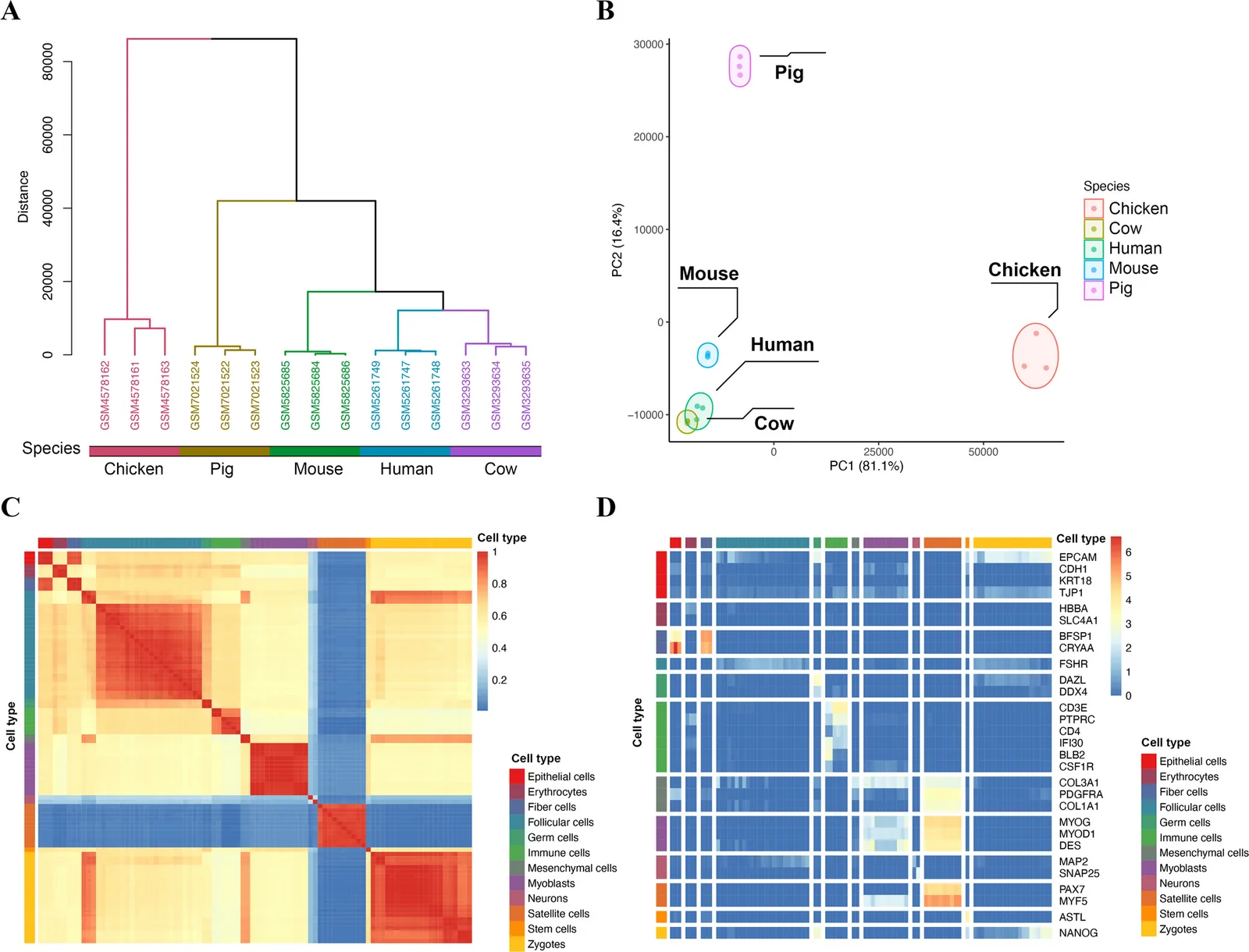
Analysis of gene expression differences among different species and construction of a reference dataset for the chicken species. **A** Cluster analysis between muscle satellite cells of different species. **B** Principal component analysis of satellite cells in muscles of different species. **C** Heat map of gene expression of different cell types in chicken species used for constructing CARD. **D** Heat map of marker gene expression in different cell types of chicken species

Based on the above analysis results (Fig. 7D and E), we observed significant differences in the gene expression profiles between chickens and other mammalian species. This observation suggests that using single-cell annotation tools developed based on mammalian data to identify chicken cell types may be insufficiently accurate, underscoring the need to establish a species-specific annotation reference dataset tailored to chickens. Since the construction of such a reference dataset typically requires bulk RNA-seq data from primary cells as a foundation, we first validated and further confirmed the substantial molecular differences between chickens and mammals in primary muscle satellite cells. Specifically, we conducted an integrative analysis of bulk RNA-seq data from humans, mice, pigs, cattle, and chickens, all of which were obtained from the GEO database. Both clustering analyses (Fig. 8A) and principal component analyses (Fig. 8B) consistently revealed that chicken satellite cells are significantly different from other mammalian species, further underscoring the necessity of developing a species-specific reference dataset for chickens.

### Construction and validation of the chicken cell annotation reference dataset (CARD)

Based on these findings, we established the first chicken primary cell annotation reference dataset—CARD. This database integrates RNA-seq data from 90 samples, covering 12 main types and 31 subtypes of primary chicken cells (Table S7). Transcriptomic analysis revealed high consistency among the same cell types (Fig. 8C), and known marker genes exhibited cell type-specific high expression patterns (Fig. 8D). These results collectively support the reliability and accuracy of the reference database.

**Fig. 9:**
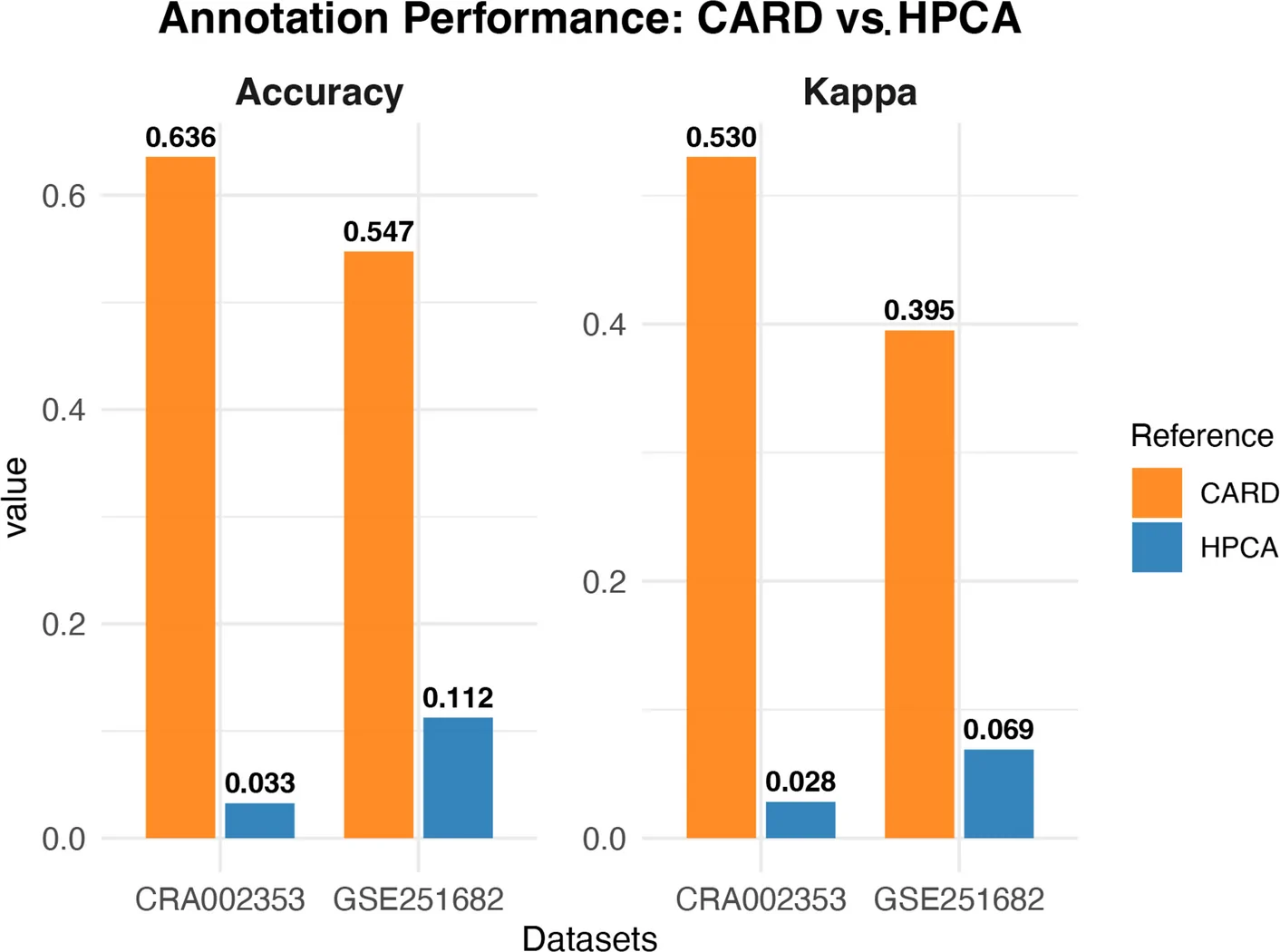
Comparison of accuracy and Kappa metrics between CARD and HPCA

To evaluate the utility of CARD, we applied it to annotate two publicly available chicken single-cell RNA-seq datasets (GSE251682 and CRA002353). According to the manual annotation of the GSE251682 data, a total of 11 cell types were identified (Fig. S9A), and the corresponding marker genes for each cell type were also highly expressed (Fig. S9B). However, using HPCA for annotation, it was found that only one endothelial cell corresponded to the manually annotated cell type (Fig. S9C). Next, using the CARD we constructed for annotation, it was found that a total of 4 cell types, such as erythrocytes, immune cells, mesenchymal cells, and myoblasts, corresponded to the manual annotation results (Fig. S9D). The same result was also found in another CRA002353 dataset, manually annotated to a total of 8 cell types (Fig. S10A and B). Using HPCA annotation, it was found that there is currently no corresponding manual annotation result (Fig. S10C). However, using CARD annotation, it was found that the four cell types of Immune cell, Myoblast, Chromocytes, and Mesenchymal cell can correspond to the results of manual annotation (Fig. S10D). To systematically evaluate the accuracy of the CARD and HPCA reference datasets in cell type annotation, we utilized public scRNA-seq datasets (CRA002353 and GSE251682) to calculate the Accuracy and Kappa metrics between the annotation results of each reference dataset and manual annotations (Fig. 9). The annotation results of the CARD dataset demonstrate an Accuracy value greater than 0.54 and a Kappa value exceeding 0.39, whereas the HPCA dataset yields an Accuracy of less than 0.12 and a Kappa value below 0.07, indicating that CARD achieves higher annotation accuracy, which confirms the importance of species-specific reference data in single-cell annotation.

Furthermore, to systematically evaluate the annotation performance of CARD on chicken single-cell transcriptomic data, this study conducted multi-dimensional validation analyses using two public datasets, CRA002353 and GSE251682. First, bubble plots of cell type-specific marker genes showed that each cell type annotated by CARD specifically expressed its corresponding canonical marker genes at high levels (Fig. S11A and B), indicating good biological rationality of the annotation results. Furthermore, representative marker genes were mapped onto the UMAP dimensionality reduction space (Fig. S12A and B), and the results revealed that cells with high expression of these genes were mainly concentrated within the corresponding cell clusters. Their spatial distribution was highly consistent with the cell types annotated by CARD, jointly supporting the reliability of the annotation results from both the aspects of clustering structure and gene expression characteristics. In addition, a Sankey diagram comparison between the CARD annotation results and manual annotations showed that the vast majority of cells in both datasets were mapped to cell types with identical or highly similar biological properties (Fig. S13A and B), demonstrating clear correspondence and high overall consistency between the two annotation approaches. Collectively, based on the validation evidence from bubble plots, UMAP spatial distributions, and Sankey diagrams, CARD can robustly identify the major cell types in both datasets, exhibiting strong generalizability and practical utility.

## Discussion

This study makes a major contribution by establishing SCAN, a large-scale single-cell database for livestock species. The platform systematically integrates a large volume of single-cell transcriptomic data from livestock, provides a set of livestock-specific marker genes, and includes custom annotation tools optimized for chickens, thus filling a key gap in livestock single-cell data resources. SCAN contains millions of cells. Compared with existing resources such as SCEA [13] and SPEED [38], SCAN has significant advantages in terms of livestock species coverage and data integration depth. In addition, a key innovation of the platform is the construction of the CARD database, which is specifically designed for non-mammalian livestock, such as chickens, whose transcriptomes differ significantly from those of mammals. This design effectively addresses the limitation of current annotation tools (such as *SingleR*), which primarily rely on reference datasets for human and mice [27]. Concurrently, SCAN integrates over 616 literature-validated species-specific cell marker genes from 6 livestock species and 1 model organism (zebrafish), effectively filling the gap in livestock data in mainstream database, such as *CellMarker* [39]. These resources collectively form a comprehensive foundation of single-cell data for livestock, significantly enhancing the accuracy of cell annotation in domestic animals and providing crucial support for in-depth, cross-species comparative and developmental studies.

Based on the SCAN platform, we performed the first single-cell resolution comparison of spermatogenesis regulatory networks between avian (chicken) and mammalian species. This analysis revealed evolutionarily conserved frameworks and significant species-specific differences. Our findings indicate that the core regulatory framework of vertebrate spermatogenesis is highly conserved evolutionarily. Early germ cells (spermatogonia and spermatocytes) exhibited similar biological properties across species, with functions primarily enriched in fundamental cellular processes such as ribonucleoprotein complex biogenesis and RNA processing. These mechanisms are critical for maintaining meiotic fidelity [40]. However, as differentiation progresses to later stages (spermatids and mature sperm), interspecies differences increase substantially, presenting a transcriptomic change pattern characterized by “early conservation, late divergence”. This pattern aligns with the perspective of evolutionary developmental biology, which posits that earlier developmental stages are subject to stronger functional constraints, while later stages are more susceptible to species-specific adaptive pressures [19, 24]. Specifically, the interspecies differences in late germ cells manifested as divergent functional strategies: mammals were significantly enriched in pathways related to sperm development and ciliary movement, whereas chickens showed strong enrichment in catabolic processes. This difference suggests that chicken sperm may rely more on metabolic energy homeostasis rather than strong autonomous motility, possibly related to their survival in the female reproductive tract [41–43]; however, this remains a hypothesis, and these observations provide preliminary clues for further exploration of the metabolic regulatory mechanisms underlying sperm survival strategies across species.

Notably, chicken-specifically expressed genes were significantly enriched in the cAMP signaling pathway. In mammals, cAMP is classically considered a key trigger for initiating sperm motility [44–46]. However, given that chicken sperm do not rely on strong autonomous motility [43], this enrichment suggests that the cAMP pathway may have additional or alternative functions in the avian reproductive system, such as participating in metabolic regulation or fertilization preparation [47]. At the cellular interaction level, we observed an extensive and uniquely active communication network between Sertoli cells and germ cells (particularly with early germ cells) in chicken testicular tissue and detected the ADGRL and DHEA signaling pathways, which are active only in chickens and are both associated with the cAMP pathway [48, 49]. Previous studies have also shown that the cAMP pathway is involved in the maturation and functional regulation of chicken Sertoli cells [47]. Taken together, these observations suggest that cAMP signaling may play a broader coordinating role in chicken spermatogenesis, although further direct evidence is needed to clarify this possibility.

Furthermore, cross-species trajectory analysis revealed further differentiation among different mammalian species during the later stages of spermatogenesis. For instance, during the SPC stage, pigs and humans showed a predominant focus on the mRNA surveillance pathway and the nucleocytoplasmic transport pathway, respectively. This difference may reflect their distinct adaptive requirements at the SPC formation stage: pigs tend to maintain a rapidly proliferating and renewing cellular state, whereas humans place greater emphasis on establishing a stable transcriptional program to ensure the precision of genetic information [50, 51].

Although SCAN provides a useful resource for cross-species comparisons, several limitations should be acknowledged. First, SCAN integrates data from different laboratories, sequencing platforms, and tissue dissociation protocols, so technical and sampling biases may exist among datasets. Second, cell type annotation relies on existing marker genes and reference datasets, and the ability to annotate rare cell types or tissues not included in the reference data remains limited. Third, although CARD outperformed HPCA on two public datasets, its coverage of cell types and sample sources is still limited. Finally, the updating and maintenance of the database depend on continuous data collection and manual curation; as new data are added, marker genes and annotation results may need further revision.

## Conclusions

In summary, this study constructed SCAN, a single-cell data platform applicable to multiple livestock species, integrating rich multi-tissue, multi-cell type data and systematic annotation tools. Taking spermatogenesis as an example, we used SCAN to identify transcriptomic differences between chicken and mammalian late spermatogenesis. These observations suggest that chickens may have a distinct metabolic strategy, though functional validation is still needed. This demonstrates the utility of SCAN in non-model species research.

In the future, the SCAN platform will focus on three key areas. First, we will regularly integrate new single-cell datasets from livestock to expand species and tissue coverage. Second, we will integrate multi-omics data, including ATAC-seq and spatial transcriptomics, to build a multimodal framework for cell typing, lineage tracing, and microenvironment analysis. Third, based on chicken reference data, we will develop species-specific annotation tools for non-model livestock to reduce annotation bias from human and mouse data. SCAN data is available free of charge for non-commercial academic use, provided the source is acknowledged; commercial use requires permission from the corresponding author.

## Supplementary Information

Additional file 1: Table S1. The data sources. Table S2. List of primers. Table S3. Statistics on the quantity and proportion of cells in testicular tissues across five species. Table S4. Conserved and species-specific expression genes across cell types in different species. Table S5. Cell–cell communication analysis in chicken testis. Table S6. List of genes associated with branch Branch 1 and Branch 2. Table S7. The data sources of the CARD datasets. Table S8. The data sources and details of the datasets.

Additional file 2: Fig. S1. Demonstration of data search and exploration in SCAN. Fig. S2. Visualization of single-cell data analysis. Fig. S3. The marker genes module. Fig. S4. Cellular composition of testis tissues across the five species. Fig. S5. Enrichment analysis of germ cells from four mammalian species. Fig. S6. Analysis of conserved and species-specific germ cell genes in testicular tissues across five species. Fig. S7. Cell–cell communication analysis across five species. Fig. S8. Pseudotime analysis of four germ cell types across five species. Fig. S9. Comparison of three cell annotation methods applied to the chicken leg muscle tissue. Fig. S10. Comparison of three cell annotation methods applied to the chicken breast muscle. Fig. S11. Cell type-specific marker gene expression profiles in CARD-annotated scRNA-seq datasets. Fig. S12. UMAP visualization of cell type-specific marker genes in CARD-annotated scRNA-seq datasets. Fig. S13. Sankey diagrams comparing CARD-based annotations with manual annotations.

## Data Availability

All scRNA-seq datasets were obtained from public databases, including GEO, ENA, and NGDC. The data accession number and link are provided in Tables S1 and S8. For user convenience, we have uploaded standardized backup datasets to the Zenodo platform (https://doi.org/10.5281/zenodo.15470864). All RNA-seq datasets were obtained from GEO databases. The data accession number and link are provided in Table S8.

## Abbreviations

ENA: European Nucleotide Archive
GEO: Gene Expression Omnibus
LCs: Leydig cells
MCs: Myoid cells
NGDC: National Genomics Data Center
SCs: Sertoli cells
SPC: Spermatocytes
SPG: Spermatogonia
SPT: Spermatids
UMIs: Unique molecular identifiers
