# Supplementary material for: SCAN: a cross-species single-cell transcriptome resource for domestic animals—revealing divergent spermatogenesis in avian and mammalian systems
Source: J Anim Sci Biotechnol. 2026 Nov 7;17:202. doi: 10.1186/s40104-026-01526-4 (PMC13744131; doi:10.1186/s40104-026-01526-4)
Supplement: Supplementary file 1 [file 40104_2026_1526_MOESM2_ESM.docx]

**Additional file 2**

**SCAN: A cross-species single-cell transcriptome resource for domestic animals — revealing divergent spermatogenesis in avian and mammalian systems**
*Yuanfang Li, Qianran Wei, Guoxi Li, Ruixing Li, Zhi Li, Xinlei Wang, Liheng Zhang, Yujie Guo, Guirong Sun, Wenting Li, Zhuanjian Li, Yadong Tian, Xiangtao Kang, Zhen Tian, Guohua Wang, Yadong Wang, Qiaoming Liu**

**This file includes:**

Supplementary Figures S1 to S13.

**Fig.S1. Demonstration of data search and exploration in SCAN.** (A) Dataset interface on the SCAN homepage; (B) Data page information panel of SCAN; (C) Visualization panel of SCAN.

**Fig.S2. Visualization of Single-Cell Data Analysis.** (A) Navigation bar of 6 modules of data visualization; (B) CellInfo and GeneExpr pages, showing cell clusters and gene expression profiles via UMAP plots; (C) Gene co-expression page, showing the distribution of two genes on UMAP or TSNE graphs at the same time; (D) Violinplot/Boxplot pages; (E) Proportion plot pages; (F) Bubbleplot/Heatmap page, displaying gene expression; (G) List of differentially expressed genes and volcano plot.

**Fig.S3. The marker genes module.** (A) Maker gene interface; (B) NCBI web page interface of maker gene; (C) PubMed interface of articles related to maker gene. (D) The UMAP and violin plots of the maker gene.

**Fig.S4. Cellular composition of testis tissues across the five species.**

**Fig.S5. Enrichment analysis of germ cells from four mammalian species.** (A) GO enrichment analysis of germ cells in human, pig, sheep, and yak, respectively; (B) KEGG enrichment analysis of germ cells in human, pig, sheep, and yak, respectively.

**Fig.S6. Analysis of conserved and species-specific germ cell genes in testicular tissues across five species.** (A) Stacked bar chart of highly expressed genes in four germ cell types across five species; (B) Venn diagram analysis of highly expressed germ cell genes across the five species; (C) GO enrichment analysis of species-specific and conserved differentially expressed germ cell genes; (D) KEGG pathway enrichment analysis of species-specific and conserved differentially expressed germ cell genes; (E) Venn diagram analysis of highly expressed genes in different germ cell types across the five species; (F) Testicular germ cell marker genes conserved in chicken species; (G) Testicular germ cell marker genes conserved in mammals; (H) GO enrichment analysis of conserved genes in different germ cell types across the five species; (I) KEGG enrichment analysis of conserved genes in different germ cell types across the five species.

**Fig.S7. Cell-Cell Communication Analysis Across Five Species.** (A-B) Analysis of the number and strength of cell-cell communication signals in human, pig, sheep, and yak; (C) The cell-cell communication signaling patterns in chicken; (D) Analysis of ADGRL and DHEA signaling networks from Sertoli cells to germ cells in chicken testis; (E) Analysis of receptor-ligand pairs in ADGRL and DHEA signaling.

**Fig.S8. Pseudotime analysis of four germ cell types across five species.**

**Fig.S9. Comparison of three cell annotation methods applied to the chicken leg muscle tissue.** (A) UMAP visualization of cell clusters from manual annotation using the public dataset GSE251682; (B) Bubble plot showing marker gene expression across cell types, including: SCs (*PAX7*, *CDH15*, *MYF5*); Myoblasts (*MYOG*, *MYOD1*); Myocytes (*ACTC1*, *MYL1*, *TPM1*, *TPM2*); Fibroblasts (*TIMP3*, *PDGFRA*, *COL1A1*, *COL1A2*); Immune cells (*IFI30*, *CSF1R*, *CD3D*, *CD3E*); Adipocytes (*ADIPOQ*); Erythrocytes (*HBBA*, *HBA1*); Neurons (*MAP2*); Endothelial cells (*CDH5*, *PECAM1*); Mesenchymal stem cells (*PDGFRA*, *COL1A1*, *COL1A2*, *COL3A1*); Glial cells (*FABP7*, *PLP1*, *MBP*); (C) Automated annotation using HPCA; (D) Automated annotation using CARD.

**Fig. S10. Comparison of three cell annotation methods applied to the chicken breast muscle.** (A) UMAP visualization of cell clusters from manual annotation using the public dataset CRA002353; (B) Bubble plot showing marker gene expression across cell types; (C) Automated annotation using HPCA; (D) Automated annotation using CARD.

**Fig. S11. Cell type-specific marker gene expression profiles in CARD-annotated scRNA-seq datasets.** (A) Bubble plot for the CRA002353 dataset; (B) Bubble plot for the GSE251682 dataset.

**Fig. S12. UMAP visualization of cell type-specific marker genes in CARD-annotated scRNA-seq datasets.** (A) CRA002353 dataset; (B) GSE251682 dataset.

**Fig. S13. Sankey diagrams comparing CARD-based annotations with manual annotations.** (A) CRA002353 dataset; (B) GSE251682 dataset.
